# Synergistic Effects of Doxorubicin and Quercetin on ROS-Associated Apoptosis and EGFR/FOXP3 Modulation in OVCAR3 Cells

**DOI:** 10.3390/biomedicines14061248

**Published:** 2026-05-30

**Authors:** Elif Ozan, Mehmet Cudi Tuncer, İlhan Özdemir

**Affiliations:** 1 Department of Gynecology and Obstetrics, Dr. Elif Ozan Practice, Ankara 06690, Turkey; drelifozan@hotmail.com; 2 Department of Anatomy, Faculty of Medicine, Dicle University, Diyarbakır 21280, Turkey; 3 Department of Histology Embryology, Faculty of Medicine, Kahramanmaraş Sütçü İmam University, Kahramanmaraş 46000, Turkey; ilhanozdemir32@hotmail.com

**Keywords:** quercetin, doxorubicin, OVCAR3, apoptosis, caspase-3, EGFR, FOXP3

## Abstract

**Background/Objectives**: Combination strategies involving natural compounds are increasingly being evaluated to improve the efficacy and safety of conventional chemotherapeutic agents. Quercetin (Q), a bioactive flavonoid, has been reported to regulate oxidative stress and apoptosis-associated signaling pathways. This study investigated whether Q enhances doxorubicin (DOX)-mediated cytotoxicity in OVCAR3 ovarian cancer cells, with particular emphasis on apoptosis, oxidative stress, and EGFR/FOXP3 signaling, while also assessing relative toxicity in HaCaT non-tumoral keratinocytes. **Methods**: Cell viability was determined using the MTT assay, and drug interactions were assessed according to the Combination Index (CI) method. Apoptosis was evaluated by Annexin V/PI flow cytometry. Caspase-3 and caspase-9 activities were measured using colorimetric assays. Intracellular reactive oxygen species (ROS) production was analyzed using the DCFH-DA assay. EGFR and FOXP3 gene expression levels were quantified by qRT-PCR, whereas caspase-9 protein expression was assessed immunocytochemically. **Results**: The DOX+Q combination produced synergistic cytotoxic effects in OVCAR3 cells (CI < 1). Compared with OVCAR3 cells, HaCaT cells displayed higher IC_50_ values following DOX treatment (7.03 µM vs. 1.42 µM) and Q treatment (183.92 µM vs. 35.94 µM), indicating relatively lower treatment sensitivity and suggesting a potentially favorable selectivity tendency; however, these findings should be regarded as preliminary. Flow cytometric findings demonstrated markedly increased proportions of both early and late apoptotic cells following combination treatment. Caspase-3 and caspase-9 activities were significantly elevated after combined exposure (*p* < 0.01). ROS production increased substantially in response to DOX+Q treatment, corresponding to an approximately 6.82-fold elevation relative to the control group. qRT-PCR analysis demonstrated reduced EGFR and FOXP3 mRNA expression levels in the combination-treated group. Immunocytochemical evaluation additionally revealed stronger caspase-9 staining intensity in treated OVCAR3 cells. **Conclusions**: These findings suggest that Q may potentiate DOX-induced cytotoxicity through mechanisms associated with enhanced oxidative stress, activation of apoptotic pathways, and modulation of proliferative signaling. The comparatively lower sensitivity observed in HaCaT cells may indicate a possible selectivity tendency; however, these observations remain preliminary and require further validation through in vivo and translational studies.

## 1. Introduction

Ovarian cancer continues to be recognized as one of the most fatal gynecological malignancies worldwide and remains a significant public health concern. It is ranked as the eighth most common cancer in women and contributes to nearly 4.7% of cancer-related mortality among the female population [1,2]. Nearly 90% of ovarian malignancies are classified as epithelial ovarian cancer, of which the serous subtype is the most frequently encountered. Although therapeutic strategies have advanced over recent decades, the five-year overall survival rate remains unsatisfactory (36–46%), even in developed countries. In comparison with malignancies such as breast cancer, these survival outcomes highlight the continuing need for improved therapeutic options [3,4,5].

DOX is a commonly utilized chemotherapeutic agent whose antitumor activity is mainly mediated through DNA intercalation and inhibition of topoisomerase II. However, its clinical application is considerably limited because of dose-dependent cardiotoxicity, which has largely been linked to ROS generation caused by iron-mediated redox cycling [6]. Accordingly, therapeutic combinations involving natural compounds have attracted growing attention as potential approaches for enhancing treatment efficacy while possibly reducing adverse effects [7,8].

Q, a naturally occurring flavonoid found abundantly in fruits and vegetables, is known to possess multiple biological properties, including antioxidant, anti-inflammatory, and anticancer activities [9]. Previous experimental findings have suggested that Q may regulate several cellular events, including induction of apoptosis, suppression of tumor proliferation, and inhibition of invasion and metastasis [10]. These biological actions have been associated with modulation of signaling pathways such as PI3K/AKT, NF-κB, p53, Wnt/β-catenin, MAPK, and JAK/STAT [10]. In ovarian cancer models, Q has been demonstrated to suppress proliferation in both dose- and time-dependent manners while promoting apoptotic responses accompanied by alterations in survival-associated proteins such as survivin [11]. Furthermore, Q has also been reported to increase intracellular accumulation of chemotherapeutic agents, including DOX, potentially through downregulation of P-glycoprotein (P-gp/ABCB1), thereby supporting its possible role as a chemosensitizing compound [12].

Variable EGFR expression has been reported in ovarian cancer and has been linked with unfavorable clinical outcomes [13]. EGFR activation contributes to tumor proliferation, survival, and metastatic progression through signaling pathways involving PI3K/AKT/mTOR and MAPK [14,15]. FOXP3, a transcription factor involved in immune regulation and tumor-associated biology, has likewise been shown to display altered expression patterns in ovarian cancer [16,17]. Therefore, the EGFR/FOXP3 axis has emerged as a potentially important molecular target, and previous investigations have suggested that this pathway may be influenced by certain combination treatments in OVCAR3 cells [18].

OVCAR3 cells are widely regarded as a clinically relevant high-grade serous ovarian cancer model and have frequently been employed in studies examining chemotherapy response and platinum resistance. Recent molecular profiling analyses demonstrated that ovarian cancer cell lines, including platinum-resistant OVCAR3-derived models, exhibit distinct platinum sensitivity characteristics associated with alterations in DNA damage response pathways, epithelial-to-mesenchymal transition, stemness-related signaling, immune/STAT activation, and regulation of drug influx-efflux mechanisms. These findings further support the translational value of OVCAR3 cells for investigating mechanisms related to therapeutic response, resistance development, and combination-based treatment strategies in ovarian cancer [19,20].

ROS serve as critical regulators of the mitochondrial apoptotic pathway and participate in programmed cell death through activation of caspase-9 and caspase-3. DOX exposure is known to elevate intracellular ROS production, whereas Q may exert antioxidant or pro-oxidant effects depending on concentration and cellular context [6,8]. This dual activity suggests that Q may influence oxidative stress responses in a way that affects chemotherapy-induced cytotoxicity. Additionally, the HaCaT cell line is commonly employed as a non-tumoral in vitro model for evaluating the relative safety profile of anticancer treatments [13].

In the present study, the cytotoxic, apoptotic, and molecular effects of combined DOX and Q treatment were investigated in OVCAR3 ovarian cancer cells, together with comparative evaluation in HaCaT cells. Cell viability, apoptosis, caspase activation, ROS generation, and expression profiles of BAX, BCL-2, NF-κB p65, EGFR, and FOXP3 were analyzed, along with immunocytochemical assessment of caspase-9 and network pharmacology-based bioinformatics analyses. We hypothesized that Q may potentiate DOX-induced cytotoxicity in OVCAR3 cells through modulation of oxidative stress-related apoptotic pathways and key signaling networks while exhibiting comparatively lower cytotoxicity in non-tumoral cells.

## 2. Materials and Methods

### 2.1. Cell Lines and Reagents

The human ovarian adenocarcinoma cell line OVCAR3 (ATCC, Manassas, VA, USA) together with the immortalized human keratinocyte cell line HaCaT (CLS, Eppelheim, Germany; catalog no. 300493) were utilized throughout the study. OVCAR3 cells were cultured in RPMI-1640 medium (Gibco, Thermo Fisher Scientific, Waltham, MA, USA) containing 20% heat-inactivated fetal bovine serum (FBS; Gibco), 0.01 mg/mL insulin (Sigma-Aldrich, St. Louis, MO, USA), 100 U/mL penicillin, and 100 µg/mL streptomycin (Sigma-Aldrich). For HaCaT cells, Dulbecco’s Modified Eagle Medium (DMEM; Gibco) supplemented with 10% FBS, 100 U/mL penicillin, and 100 µg/mL streptomycin was used as the culture medium.

Cell cultures were incubated under humidified conditions at 37 °C with 5% CO_2_. Fresh culture medium was supplied every 2–3 days, and subculturing was performed when cell confluence reached approximately 70–80% using trypsin-EDTA solution (Gibco).

DOX and Q were obtained from Sigma-Aldrich (St. Louis, MO, USA). Dimethyl sulfoxide (DMSO; Sigma-Aldrich) was used for preparation of stock solutions, which were subsequently diluted in culture medium to the required working concentrations immediately before treatment applications. In all experimental groups, the final DMSO concentration remained below 0.1% (*v*/*v*).

### 2.2. Cell Viability and Combination Analysis

Cell viability analysis was performed using the colorimetric 3-(4,5-dimethylthiazol-2-yl)-2,5-diphenyltetrazolium bromide assay (MTT; Sigma-Aldrich, St. Louis, MO, USA). OVCAR3 and HaCaT cells were plated into 96-well culture plates at a density of 5 × 10^3^ cells/well and maintained under standard culture conditions for 24 h to allow cellular attachment.

After the attachment period, cells were exposed to increasing concentrations of DOX (0.5–25 µM) and Q (5–200 µM) for 24 and 48 h. At the end of treatment, MTT reagent (0.5 mg/mL) was added to each well, followed by incubation at 37 °C for 4 h. Formazan crystals generated during incubation were solubilized with dimethyl sulfoxide (DMSO), and absorbance values were recorded at 570 nm using 630 nm as the reference wavelength with a microplate reader (Multiskan GO, Thermo Fisher Scientific, Waltham, MA, USA).

Relative cell viability was calculated as a percentage of untreated control cells according to the following equation: (absorbance of treated cells/absorbance of control cells) × 100. Dose–response plots were constructed, and IC_50_ values were determined by nonlinear regression analysis using GraphPad Prism 9.0 software (GraphPad Software, San Diego, CA, USA).

For combination experiments, 48 h IC_50_ values were selected as operational reference points for the establishment of fixed-ratio treatment designs. DOX and Q combinations were prepared at 1:10, 1:20, and 1:50 ratios, and complete dose–response matrices were generated using multiple concentration levels corresponding to 0.5×, 1×, 2×, 4×, and 8× IC_50_ values for both individual treatments and combined applications.

Interactions between drugs were analyzed according to the Chou–Talalay method using CompuSyn software (Version 1.0; ComboSyn Inc., Paramus, NJ, USA). CI values were calculated based on the median-effect principle, where CI < 1 represented synergism, CI = 1 indicated an additive interaction, and CI > 1 reflected antagonism. All experiments were repeated in at least three independent experiments, with each experiment performed in triplicate wells.

### 2.3. Apoptosis Analysis by Flow Cytometry

Apoptosis was evaluated by Annexin V-FITC/propidium iodide (PI) dual staining in combination with flow cytometric analysis. OVCAR3 and HaCaT cells were plated in 6-well plates at a density of 3 × 10^5^ cells per well and incubated overnight to allow attachment. After incubation, cells were exposed for 48 h to DOX, Q, or combined treatment at concentrations corresponding to the respective 48 h IC_50_ values.

At the end of treatment, adherent and floating cells were harvested together and washed twice with cold phosphate-buffered saline (PBS). Cell pellets were then resuspended in Annexin V binding buffer (10 mM HEPES, pH 7.4; 140 mM NaCl; 2.5 mM CaCl_2_) to obtain a final concentration of 1 × 10^6^ cells/mL. Subsequently, cells were stained with 5 µL FITC-conjugated Annexin V and 5 µL PI (BD Biosciences Pharmingen, San Jose, CA, USA) and incubated for 15 min at room temperature under dark conditions.

Flow cytometric acquisition was carried out using a BD FACSCanto II flow cytometer (BD Biosciences, San Jose, CA, USA), and FlowJo software (v10, BD Life Sciences) was used for data analysis. A minimum of 10,000 events was collected for each sample. Debris and doublets were excluded through gating procedures based on forward- and side-scatter characteristics.

To reduce possible interference related to DOX-associated autofluorescence and spectral overlap, unstained controls, single-stained controls, fluorescence-minus-one controls, and fluorescence compensation settings were incorporated during acquisition and analytical procedures (Supplementary Table S1). Prior to fluorescence-based population assessment, gating strategies were established using FSC/SSC discrimination together with singlet selection.

Cell populations were categorized as viable (Annexin V^−^/PI^−^), early apoptotic (Annexin V^+^/PI^−^), late apoptotic/necrotic (Annexin V^+^/PI^+^), and necrotic (Annexin V^−^/PI^+^). Annexin V^+^/PI^+^ cells were collectively interpreted as late apoptotic/secondary necrotic populations rather than completely separate biological entities. Percentages of all populations were quantified and included in subsequent analyses. Each experiment was repeated in at least three independent biological replicates.

### 2.4. Caspase Activity Assays

Caspase-3 and caspase-9 enzymatic activities were determined using commercially available colorimetric assay kits (Caspase-3 Colorimetric Assay Kit; Abcam, Cambridge, UK; Cat. No. ab39401 and Caspase-9 Colorimetric Assay Kit; R&D Systems, Minneapolis, MN, USA; Cat. No. K119100) in accordance with the manufacturers’ protocols. OVCAR3 cells were exposed for 48 h to DOX, Q, or combined treatment at concentrations corresponding to the respective 48 h IC_50_ values.

The fixed DOX:Q ratios selected for combination treatment (1:10, 1:20, and 1:50) were established according to preliminary IC_50_ screening data and the relative responsiveness of OVCAR3 cells to each compound. These ratios were applied to allow systematic assessment of synergistic, additive, or antagonistic interactions at different effect levels using the Chou–Talalay combination index method.

After treatment, cells were collected and lysed using the lysis buffer supplied with the assay kits. Cytosolic protein fractions were obtained by centrifugation of the lysates at 10,000× *g* for 15 min at 4 °C. Protein concentrations were quantified with the Bradford assay (Bio-Rad Laboratories, Hercules, CA, USA). To maintain consistency among samples, equal protein amounts (typically 100–200 µg per reaction) were used in all assays.

Caspase-3 activity was assessed using the DEVD-pNA substrate, whereas caspase-9 activity was evaluated with the LEHD-pNA substrate. Following incubation with the appropriate substrates at 37 °C for the recommended duration, released p-nitroaniline (pNA) levels were determined by absorbance measurement at 405 nm using a microplate reader. Enzyme activities were calculated according to pNA standard curves and reported as nmol pNA/min/mg protein. All assays were carried out in at least three independent biological replicates, with duplicate or triplicate measurements performed for each replicate.

### 2.5. Intracellular ROS Measurement

Intracellular ROS production was evaluated using the fluorescent probe 2′,7′-dichlorodihydrofluorescein diacetate (DCFH-DA; Sigma-Aldrich, St. Louis, MO, USA; Cat. No. D6883). OVCAR3 cells were exposed to DOX, Q, or combined treatment at concentrations corresponding to the respective 48 h IC_50_ values for 48 h.

After treatment exposure, cells were incubated with 10 µM DCFH-DA for 30 min at 37 °C under dark conditions. Subsequently, excess dye was removed by washing cells twice with cold PBS, after which cells were detached using trypsin-EDTA. Cell suspensions were then collected and subjected to immediate analysis.

Fluorescence measurements were performed using a BD FACSCanto II flow cytometer (BD Biosciences, San Jose, CA, USA) in the FITC detection channel (excitation: 488 nm, emission: 525 nm). For each sample, at least 10,000 events were acquired. Debris and cellular aggregates were excluded through gating based on forward- and side-scatter parameters.

Since intracellular ROS detection relied on DCFH-DA fluorescence measured in the FITC channel, additional unstained controls, autofluorescence controls, and fluorescence compensation procedures were incorporated during acquisition and analysis to reduce potential signal interference related to the intrinsic fluorescence characteristics of DOX (Supplementary Table S1).

ROS production was expressed as mean fluorescence intensity (MFI) normalized against the control group. Data were reported as fold changes relative to untreated cells. All experiments were conducted using at least three independent biological replicates.

### 2.6. NAC Rescue Assay

To investigate the possible contribution of ROS to apoptosis induction, a pretreatment protocol using N-acetyl-L-cysteine (NAC; Sigma-Aldrich, St. Louis, MO, USA) was applied. Prior to exposure to the DOX and Q combination at concentrations corresponding to the 48 h IC_50_ values, OVCAR3 cells were incubated with 5 mM NAC for 2 h.

Following treatment procedures, caspase-3 and caspase-9 activities were determined as described previously. Activity levels measured in the NAC+DOX+Q group were subsequently compared with those obtained from the DOX+Q treatment group without NAC pretreatment.

Decreased caspase activity observed after NAC pretreatment was interpreted as suggesting a possible involvement of ROS in the apoptotic process rather than conclusive evidence for a single mechanistic pathway.

All experiments were conducted using at least three independent biological replicates. Results were presented as mean ± standard deviation (SD). Statistical analyses among groups were performed by one-way analysis of variance (ANOVA) followed by Tukey’s post hoc test, and *p* < 0.05 was accepted as statistically significant.

### 2.7. Gene Expression Analysis by RT-qPCR

mRNA expression levels of BAX, BCL-2, NF-κB p65 (RELA), EGFR, and FOXP3 in OVCAR3 cells were analyzed by quantitative real-time polymerase chain reaction (RT-qPCR).

Total RNA isolation was carried out using TRIzol reagent (Invitrogen, Thermo Fisher Scientific, Waltham, MA, USA) according to the manufacturer’s recommendations. RNA quantity and purity were evaluated with a NanoDrop 2000 spectrophotometer (Thermo Fisher Scientific), and samples presenting A260/A280 ratios within the range of 1.8–2.0 were accepted for further analysis.

For cDNA synthesis, 1 µg of total RNA was reverse-transcribed using the High Capacity cDNA Reverse Transcription Kit (Applied Biosystems, Thermo Fisher Scientific) in accordance with the manufacturer’s protocol.

Amplification reactions for RT-qPCR were conducted using a 7500 Fast Real-Time PCR System (Applied Biosystems, Foster City, CA, USA) together with SYBR Green Master Mix (Applied Biosystems) and gene-specific primer sets. Thermal cycling parameters included an initial denaturation step at 95 °C for 10 min, followed by 40 amplification cycles consisting of denaturation at 95 °C for 15 s and annealing/extension at 60 °C for 1 min.

Primer amplification efficiencies, determined by standard curve analysis, ranged from 90% to 110% for all primer pairs. Specificity of amplification was verified through melting curve analysis, which confirmed the presence of a single amplification product for each target gene.

Relative mRNA expression levels were determined using the 2^−ΔΔCt^ method, with GAPDH serving as the endogenous reference gene for normalization. All analyses were performed using three independent biological replicates, each evaluated in technical triplicates. Primer sequences are listed in Table 1.

### 2.8. Immunocytochemistry

Immunocytochemical staining was performed to assess caspase-9 protein expression. OVCAR3 cells were plated onto chamber slides and exposed for 48 h to DOX, Q, or combined treatment at concentrations corresponding to the respective 48 h IC_50_ values.

After treatment, fixation was carried out using 4% paraformaldehyde (PFA) for 15 min at room temperature. Samples were subsequently washed three times with PBS containing 0.1% Tween-20 (PBS-T). To reduce non-specific antibody binding, cells were incubated with 5% normal goat serum for 1 h at room temperature.

Thereafter, cells were incubated overnight at 4 °C with a primary antibody directed against caspase-9 (Cell Signaling Technology, Danvers, MA, USA; 1:200 dilution). Following PBS-T washing steps, samples were treated for 1 h at room temperature with a horseradish peroxidase (HRP)-linked secondary antibody (Abcam, Cambridge, UK; 1:500 dilution).

Signal visualization was achieved using 3,3′-diaminobenzidine (DAB; Dako, Agilent Technologies, Santa Clara, CA, USA) as the chromogenic substrate, followed by hematoxylin counterstaining. Microscopic examination was performed using an Olympus BX53 light microscope (Olympus, Tokyo, Japan), and representative images were obtained under identical exposure settings.

Semi-quantitative assessment of staining intensity was conducted using ImageJ software (Version 1.54; NIH, Bethesda, MD, USA). H-score calculations were performed according to the following formula:

H-score = [1 × (% weak staining) + 2 × (% moderate staining) + 3 × (% strong staining)],

resulting in a total score between 0 and 300.

All evaluations were independently carried out by two investigators blinded to the treatment groups. Quantification included assessment of at least 10 randomly selected microscopic fields per group, corresponding to a minimum of 100 analyzed cells. All experiments were repeated in at least three independent biological replicates.

### 2.9. Network Pharmacology and Bioinformatics Analysis

To investigate potential molecular targets and signaling pathways associated with the combined effects of DOX and Q in ovarian cancer, a network pharmacology-based bioinformatics analysis was performed.

**Target Identification:** Targets potentially associated with Q were identified using the SwissTargetPrediction (http://www.swisstargetprediction.ch/, accessed on 10 March 2026), TCMSP (https://tcmsp-e.com/tcmsp.php?sf=1, accessed on 10 March 2026), and PharmMapper (http://www.lilab-ecust.cn/pharmmapper/, accessed on 10 March 2026) databases. Targets related to DOX were collected from DrugBank (https://go.drugbank.com/, accessed on 10 March 2026) and ChEMBL (https://www.ebi.ac.uk/chembl/, accessed on 10 March 2026). Genes associated with ovarian cancer were retrieved from GeneCards (https://www.genecards.org/, accessed on 10 March 2026), DisGeNET (https://www.disgenet.org/, accessed on 10 March 2026), and OMIM (https://omim.org/, accessed on 10 March 2026).

To enhance dataset consistency, duplicate records were eliminated and gene nomenclature was standardized according to UniProt (https://www.uniprot.org/, accessed on 10 March 2026). Overlapping targets between drug-associated datasets and ovarian cancer–related genes were subsequently identified and visualized using the Venn diagram platform (https://bioinformatics.psb.ugent.be/webtools/Venn/, accessed on 10 March 2026). Genes located within the intersection set were defined as potential targets related to the DOX+Q combination.

**Protein–Protein Interaction (PPI) Network Analysis:** The intersecting genes were uploaded to the STRING database (https://string-db.org/, accessed on 10 March 2026) for construction of a protein–protein interaction (PPI) network. Analyses were limited to Homo sapiens, and the minimum interaction confidence score was set at ≥0.700 to ensure high-confidence interactions. The generated network was subsequently subjected to further evaluation using Cytoscape software (version 3.10.0; https://cytoscape.org/, accessed on 10 March 2026). Identification of hub genes was performed with the CytoHubba plugin (v0.1) based on topological features including degree, betweenness centrality, and closeness centrality. In network visualizations, node sizes were adjusted according to degree values.

**GO and KEGG Enrichment Analysis:** Gene ontology (GO) enrichment analyses covering biological process (BP), molecular function (MF), and cellular component (CC) categories, together with Kyoto Encyclopedia of Genes and Genomes (KEGG) pathway analysis, were carried out using the DAVID database (https://davidbioinformatics.nih.gov/, accessed on 10 March 2026) and the Metascape platform (https://metascape.org/, accessed on 10 March 2026). Significantly enriched terms were identified using thresholds of *p* < 0.05 and false discovery rate (FDR) < 0.05. Visualization of enrichment outputs was performed with bubble plots generated through the bioinformatics.com.cn platform (accessed on 10 March 2026).

This bioinformatics approach was applied to obtain a broader systems-level understanding of potential molecular interactions; accordingly, the resulting findings should be interpreted as hypothesis-generating observations rather than definitive mechanistic evidence.

### 2.10. Statistical Analysis

All experimental results are presented as mean ± standard deviation (SD) and were obtained from a minimum of three independent biological replicates.

For analyses of cell viability, caspase activity, ROS production, and gene expression, comparisons among experimental groups were carried out using one-way analysis of variance (ANOVA) followed by Tukey’s post hoc multiple comparison test.

In flow cytometry–based apoptosis experiments, the percentages of viable, early apoptotic, late apoptotic/necrotic, and necrotic cell populations were quantified and statistically evaluated by one-way ANOVA with Tukey’s post hoc analysis.

For RT-qPCR experiments, statistical analyses were performed using ΔCt values, whereas relative gene expression levels were presented according to the 2^−ΔΔCt^ calculation method.

The interaction between DOX and Q was analyzed using the Chou–Talalay method with CompuSyn software (ComboSyn Inc., Paramus, NJ, USA), and findings were reported as CI values. Interpretation of CI values was descriptive in nature (CI < 1: synergism; CI = 1: additive effect; CI > 1: antagonism) and no inferential statistical analysis was applied to these data. Prior to statistical testing, assumptions of normality were taken into consideration. When pairwise analyses were specifically necessary, Student’s *t*-test was employed. All statistical procedures were performed using GraphPad Prism 9.0 software (GraphPad Software, San Diego, CA, USA), and statistical significance was defined as *p* < 0.05.

## 3. Results

### 3.1. Effects of DOX and Q on Cell Viability in OVCAR3 and HaCaT Cells

**Figure 1:**
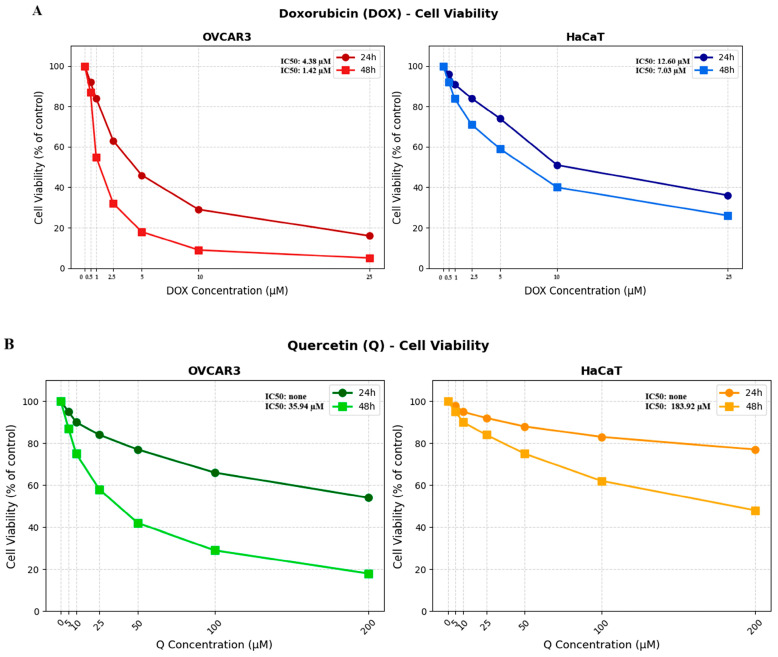
Effects of DOX and Q on cell viability in OVCAR3 and HaCaT cells. Cells were treated with increasing concentrations of DOX (0–25 µM) and Q (0–200 µM) for 24 and 48 h. Cell viability was assessed by MTT assay and expressed as percentage relative to untreated control cells (mean ± SD, n = 3). Statistical analysis was performed using one-way ANOVA followed by Tukey’s post hoc test. (**A**) DOX treatment resulted in a dose- and time-dependent decrease in cell viability in both OVCAR3 and HaCaT cells. IC_50_ values were calculated as 4.38 µM (24 h) and 1.42 µM (48 h) in OVCAR3 cells, and 12.60 µM (24 h) and 7.03 µM (48 h) in HaCaT cells. (**B**) Q treatment showed a time-dependent reduction in cell viability. In OVCAR3 cells, IC_50_ values were not reached at 24 h and were calculated as 35.94 µM at 48 h. In HaCaT cells, IC_50_ values were not reached at 24 h and were calculated as 183.92 µM at 48 h.

DOX treatment produced a concentration- and time-dependent decline in OVCAR3 cell viability (Figure 1A). After 24 h of exposure, viability markedly decreased at concentrations ≥5 µM, and values fell below 50% at higher doses. A stronger cytotoxic response was observed at 48 h, where reduced viability became apparent beginning at concentrations ≥1 µM, indicating enhanced time-dependent activity. Nonlinear regression analysis identified IC_50_ values of 4.38 µM at 24 h and 1.42 µM at 48 h.

Compared with OVCAR3 cells, HaCaT cells exhibited a less pronounced reduction in viability following DOX exposure (Figure 1A). At 24 h, substantial viability loss was mainly detected at the highest tested concentration (25 µM). Following 48 h treatment, viability decreased below 50% within the 10–25 µM concentration range. Calculated IC_50_ values were 12.60 µM at 24 h and 7.03 µM at 48 h, suggesting lower DOX sensitivity in HaCaT cells relative to OVCAR3 cells.

Exposure to Q induced time-dependent alterations in cell viability (Figure 1B). In OVCAR3 cells, viability remained above 50% at all tested concentrations after 24 h treatment; therefore, an IC_50_ value could not be determined at this time point. Following 48 h exposure, the calculated IC_50_ value was 35.94 µM.

In HaCaT cells, Q exposure produced only limited cytotoxic effects at 24 h, with viability remaining above 70% throughout all tested concentrations. At 48 h, a moderate decrease in viability was detected, and the IC_50_ value was determined as 183.92 µM. Collectively, these findings indicate comparatively lower sensitivity of HaCaT cells to Q treatment relative to OVCAR3 cells; however, these observations should be considered preliminary and interpreted cautiously.

### 3.2. Effects of NAC Pretreatment on DOX- and Q-Induced Changes in Cell Viability in OVCAR3 Cells

To investigate the involvement of ROS in treatment-associated cytotoxicity, OVCAR3 cells were incubated with 5 mM NAC for 2 h before treatment with DOX, Q, or the DOX+Q combination at concentrations corresponding to the respective 48 h IC_50_ values. Cell viability was then determined using the MTT assay.

**Figure 2:**
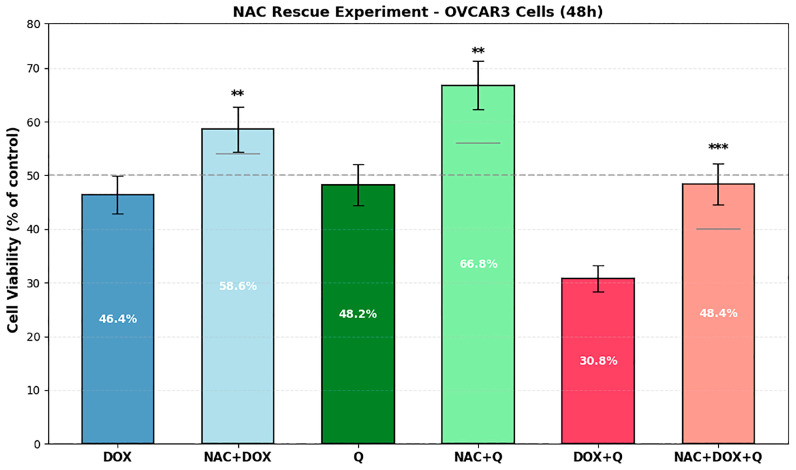
Effects of NAC pretreatment on cell viability in OVCAR3 cells. OVCAR3 cells were pretreated with 5 mM NAC for 2 h prior to treatment with DOX (DOX; 1.42 µM), Q (Q; 35.94 µM), or their combination for 48 h. Cell viability was assessed using the MTT assay and expressed as a percentage relative to untreated control cells (mean ± SD, n = 3). NAC pretreatment increased cell viability in DOX-, Q-, and DOX+Q-treated groups compared with the corresponding treatments without NAC. Statistical analysis was performed using one-way ANOVA followed by Tukey’s post hoc test. Statistical significance is indicated as ** *p* < 0.01 and *** *p* < 0.001 versus the corresponding treatment without NAC.

As presented in Figure 2, NAC treatment alone produced no substantial change in viability (96.8 ± 4.1% relative to control). Exposure to DOX reduced viability to 46.4 ± 3.5%, whereas pretreatment with NAC increased viability to 58.6 ± 4.2% (*p* < 0.01 vs. DOX alone). Likewise, viability decreased to 48.2 ± 3.8% following Q treatment, while NAC pretreatment increased this value to 66.8 ± 4.5% (*p* < 0.01 vs. Q alone).

Among all treatment groups, the DOX+Q combination produced the greatest reduction in viability, decreasing it to 30.8 ± 2.5%. Following NAC pretreatment, viability in the combination group increased to 48.4 ± 3.8% (*p* < 0.001 vs. DOX+Q).

Overall, pretreatment with NAC was associated with partial recovery of cell viability in all treatment conditions. These observations suggest that oxidative stress may participate in the cytotoxic response, although it does not appear to be the only contributing mechanism.

### 3.3. Synergistic Interaction Between DOX and Q in OVCAR3 Cells Based on Combination Index and Isobologram Analysis

The interaction between DOX and Q in OVCAR3 cells was evaluated using the Chou–Talalay method across multiple concentration combinations at 48 h.

**Figure 3:**
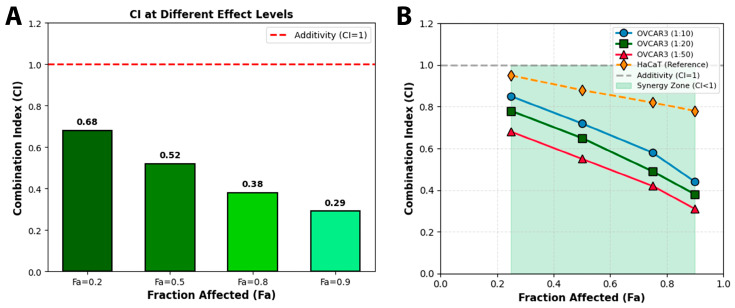
CI analysis of DOX and Q in OVCAR3 cells. Cells were treated with DOX and Q at fixed concentration ratios for 48 h. Combination ratios (1:10, 1:20, and 1:50) were selected based on relative IC_50_ values and fixed-ratio combination design principles commonly used in CI-based synergy analyses. CI values were calculated using the Chou–Talalay method based on the median-effect principle. Data were derived from three independent biological replicates (n = 3). (**A**) CI values plotted as a function of fraction affected (Fa). CI values ranged from 0.68 to 0.29 across Fa levels of 0.25 to 0.90. (**B**) CI values calculated at fixed concentration ratios (1:10, 1:20, and 1:50) across multiple Fa levels (0.25, 0.50, 0.75, and 0.90). Values below 1 indicate synergistic interaction.

CI analysis demonstrated that the DOX+Q combination exerted predominantly synergistic effects across a wide range of tested concentrations (Figure 3A). CI values were consistently below 1 at multiple fraction affected (Fa) levels, indicating synergistic interactions between the two agents.

Isobologram analysis further supported these findings (Figure 3B). The majority of combination data points corresponding to Fa levels of 0.5, 0.75, and 0.9 were located below the line of additivity, confirming synergistic interactions between DOX and Q. Notably, the deviation from the additivity line became more pronounced at higher Fa levels, suggesting an increased degree of synergy at higher levels of growth inhibition.

These results indicate that the combination of DOX and Q produces a synergistic reduction in cell viability in OVCAR3 cells under the tested conditions.

**Figure 4:**
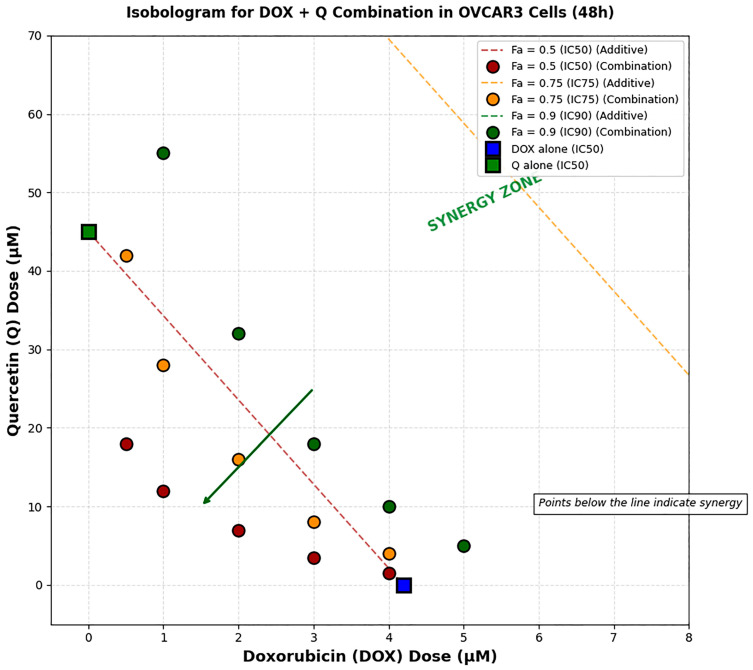
Isobologram analysis of DOX and Q combination in OVCAR3 cells. Cells were treated with DOX and Q alone or in combination at fixed concentration ratios for 48 h. The isobologram illustrates the dose combinations required to achieve fraction affected (Fa) levels of 0.5, 0.75, and 0.9. The diagonal lines represent the theoretical additive effect at each Fa level. Experimental combination points (closed symbols) located below the additive lines indicate synergistic interaction, whereas points above the lines would indicate antagonism. The green arrow indicates the direction toward the synergy region, where experimental combination points located below the theoretical additivity line reflect synergistic interactions between DOX and Q. In this analysis, combination data points were predominantly located below the additive lines across all tested Fa levels, consistent with a synergistic interaction between DOX and Q in OVCAR3 cells. Data are representative of three independent biological replicates (n = 3).

According to the isobologram analysis, most combination data points were positioned below the additivity line (Figure 4). This distribution pattern supports a synergistic interaction between DOX and Q, suggesting that similar cytotoxic responses were achieved with lower concentrations compared with single-agent exposure.

### 3.4. Effects of DOX and Q on Apoptotic Cell Death in OVCAR3 Cells

**Figure 5:**
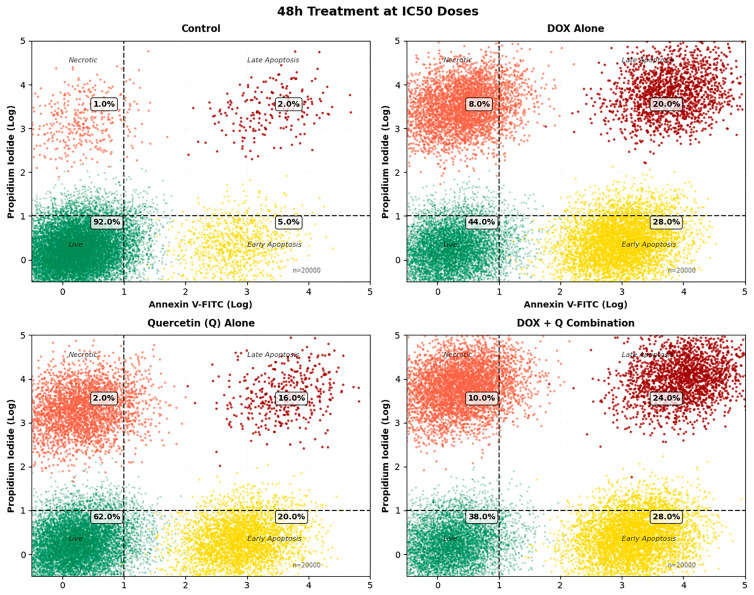
Apoptosis analysis of DOX, Q, and their combination in OVCAR3 cells by flow cytometry. OVCAR3 cells were treated with DOX, Q, or their combination at concentrations corresponding to their 48 h IC_50_ values. Apoptosis was assessed by Annexin V-FITC/PI staining followed by flow cytometry. Representative dot plots show the distribution of viable (Annexin V^−^/PI^−^), early apoptotic (Annexin V^+^/PI^−^), late apoptotic/necrotic (Annexin V^+^/PI^+^), and necrotic (Annexin V^−^/PI^+^) cell populations. The combination treatment was associated with an increased proportion of total apoptotic cells (early + late apoptosis) compared with single-agent treatments. Data are presented as mean ± SD from three independent biological replicates (≥20,000 events per sample, n = 3). Statistical analysis was performed using one-way ANOVA followed by Tukey’s post hoc test. Flow cytometric acquisition and compensation parameters are provided in Supplementary Table S1.

Annexin V-FITC/PI flow cytometric analysis was performed to assess apoptotic cell death in OVCAR3 cells after 48 h exposure to DOX, Q, and the DOX+Q combination using the FITC Annexin V Apoptosis Detection Kit I (BD Biosciences Pharmingen, San Jose, CA, USA; Cat. No. 556547) (Figure 5).

In the control group, most cells remained viable (92%), whereas only minor proportions of early apoptotic (5%) and late apoptotic (2%) cells were detected. Following DOX treatment, the viable cell fraction decreased markedly to 44%, together with increases in early apoptotic (28%) and late apoptotic/necrotic (20%) populations.

Treatment with Q alone produced a moderate reduction in viability, with 62% of cells remaining viable. Early apoptotic and late apoptotic/necrotic populations accounted for 20% and 16% of the cells, respectively.

The DOX+Q combination caused a further decline in viable cells to 38%. While the proportion of early apoptotic cells (28%) remained similar to that observed following DOX treatment alone, the late apoptotic/necrotic population increased to 24%. In addition, necrotic cells showed a slight elevation in the combination-treated group (10%) compared with single-agent treatments.

Overall, combination treatment was associated with reduced cell viability together with a shift toward higher late apoptotic/necrotic cell populations.

### 3.5. Effects of DOX and Q on Caspase-3 and Caspase-9 Activities in OVCAR3 Cells

**Figure 6:**
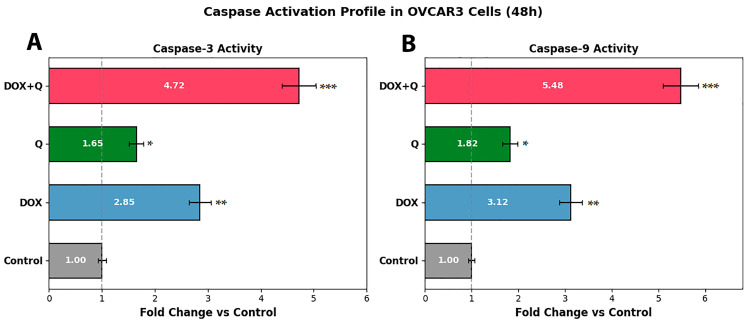
Caspase-3 and caspase-9 activities in OVCAR3 cells following treatment with DOX, Q, and their combination. OVCAR3 cells were treated with DOX, Q, or their combination at concentrations corresponding to their 48 h IC_50_ values. Data are presented as mean ± SD from three independent biological replicates (n = 3). (**A**) Caspase-3 activity expressed as fold change relative to control. DOX and Q treatments increased caspase-3 activity to 2.85-fold and 1.65-fold, respectively, while the combination treatment resulted in a 4.72-fold increase. (**B**) Caspase-9 activity expressed as fold change relative to control. DOX and Q treatments increased caspase-9 activity to 3.12-fold and 1.82-fold, respectively, whereas the combination treatment resulted in a 5.48-fold increase. Statistical significance is indicated as * *p* < 0.05, ** *p* < 0.01, and *** *p* < 0.001 versus control (one-way ANOVA followed by Tukey’s post hoc test).

Following 48 h exposure to DOX, Q, or combined treatment, caspase-3 and caspase-9 activities were analyzed in OVCAR3 cells (Figure 6).

Relative to the control group (1.00), caspase-3 activity increased 2.85-fold after DOX treatment and 1.65-fold after Q treatment. The highest elevation was observed in the DOX+Q group, where caspase-3 activity reached 4.72-fold compared with control values.

A comparable trend was detected for caspase-9 activity. Treatment with DOX increased caspase-9 activity to 3.12-fold, whereas Q treatment resulted in a 1.82-fold increase relative to control. The combination group demonstrated the greatest increase, reaching 5.48-fold above control levels.

In summary, combined DOX+Q treatment was associated with greater caspase-3 and caspase-9 activation than either single-agent treatment alone.

### 3.6. Effects of DOX and Q on Intracellular ROS Levels in OVCAR3 Cells

**Figure 7:**
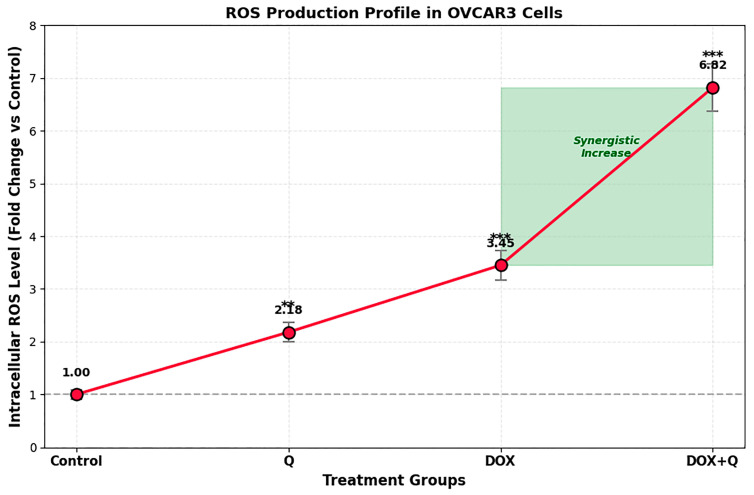
Intracellular ROS levels in OVCAR3 cells following treatment with DOX, Q, and their combination. OVCAR3 cells were treated with DOX, Q, or their combination for 48 h. Intracellular ROS levels were assessed using the DCFH-DA fluorescent probe followed by flow cytometric analysis. Fluorescence intensity was measured in the FITC channel (excitation: 488 nm, emission: 525 nm), and ROS levels were expressed as fold change relative to control (mean ± SD, n = 3). Statistical analysis was performed using one-way ANOVA followed by Tukey’s post hoc test. Statistical significance is indicated as ** *p* < 0.01, and *** *p* < 0.001 versus control. Flow cytometric acquisition and compensation parameters are provided in Supplementary Table S1.

DCFH-DA–based flow cytometric analysis was performed to determine intracellular ROS levels after 48 h treatment with DOX, Q, and the DOX+Q combination (Figure 7).

Compared with the control group (1.00), ROS production increased 2.18-fold following Q treatment and 3.45-fold following DOX treatment.

Among all experimental groups, the highest ROS level was detected in cells treated with the DOX+Q combination, reaching a 6.82-fold increase relative to control values. This increase was significantly greater than those observed with either single-agent treatment alone (*p* < 0.001).

Overall, combined DOX and Q exposure was associated with a pronounced elevation in intracellular ROS generation compared with treatment using DOX or Q alone.

### 3.7. Effects of NAC Pretreatment on Intracellular ROS Levels in OVCAR3 Cells

**Figure 8:**
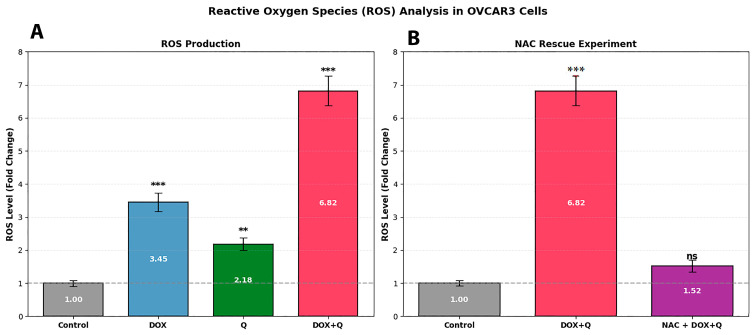
Effects of NAC pretreatment on intracellular ROS levels in OVCAR3 cells following DOX and Q treatment. (**A**) Intracellular ROS production in OVCAR3 cells treated with DOX, Q, and their combination (DOX+Q) at concentrations corresponding to their 48 h IC_50_ values. ROS levels were measured using the DCFH-DA fluorescence assay. The DOX+Q combination markedly increased ROS levels (6.82-fold) compared with control (1.00), while DOX and Q alone induced moderate increases (3.45-fold and 2.18-fold, respectively). (**B**) NAC rescue experiment. Cells were pretreated with 5 mM NAC for 2 h prior to DOX+Q co-treatment. NAC pretreatment reduced ROS levels from 6.82-fold (DOX+Q) to 1.52-fold, consistent with attenuation of oxidative stress. Data are presented as mean ± SD (n = 3). Statistical significance is indicated as ** *p* < 0.01 and *** *p* < 0.001 versus control (one-way ANOVA followed by Tukey’s post hoc test). ns, not significant versus control.

To further investigate the involvement of ROS in the observed cellular effects, intracellular ROS production was analyzed in OVCAR3 cells following NAC pretreatment (Figure 8).

Exposure to the DOX+Q combination increased ROS levels to 6.82-fold relative to the control group. Following NAC pretreatment, ROS levels decreased markedly to 1.52-fold, representing a significant reduction compared with the DOX+Q-treated group (*p* < 0.001).

Although NAC pretreatment substantially reduced ROS accumulation, ROS values did not completely return to baseline control levels. Overall, pretreatment with NAC was associated with a pronounced attenuation of ROS elevation induced by the combination treatment.

### 3.8. Effects of DOX and Q on Caspase-9 Protein Expression in OVCAR3 Cells

**Figure 9:**
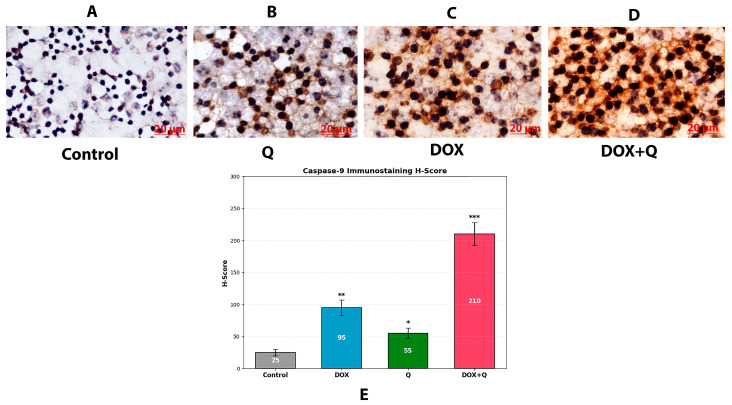
Immunocytochemical assessment of caspase-9 immunoreactivity in OVCAR3 cells following treatment with DOX, Q, and their combination. OVCAR3 cells were treated with DOX, Q, or their combination at concentrations corresponding to their 48 h IC_50_ values for 48 h. (**A**–**D**) Representative light microscopy images (200× magnification) showing caspase-9 immunostaining in control (**A**), Q-treated (**B**), DOX-treated (**C**), and DOX+Q-treated (**D**) cells. Scale bar = 20 µm. (**E**) Semi-quantitative analysis of caspase-9 immunoreactivity based on H-score evaluation. Data are presented as mean ± SD (n = 3). Statistical significance is indicated as * *p* < 0.05, ** *p* < 0.01, and *** *p* < 0.001 versus control (one-way ANOVA followed by Tukey’s post hoc test).

Immunocytochemical analysis was performed to determine caspase-9 protein expression in OVCAR3 cells after treatment with DOX, Q, and the DOX+Q combination (Figure 9).

Only minimal caspase-9 immunoreactivity with weak cytoplasmic staining was observed in control cells. Treatment with Q produced a moderate increase in staining intensity (H-score: 55), whereas a more pronounced increase was detected following DOX treatment (H-score: 95).

Among all treatment groups, the DOX+Q combination exhibited the strongest caspase-9 immunoreactivity, characterized by intense and diffuse cytoplasmic staining (H-score: 210).

This staining pattern was further supported by semi-quantitative H-score analysis performed with ImageJ software, which demonstrated a stepwise increase in caspase-9 expression across the treatment groups (control < Q < DOX < DOX+Q).

Overall, the combination treatment was associated with greater caspase-9 expression compared with either single-agent treatment alone.

### 3.9. Effects of DOX and Q on Apoptosis-, Inflammation-, and Growth-Related Gene Expression in OVCAR3 Cells

**Figure 10:**
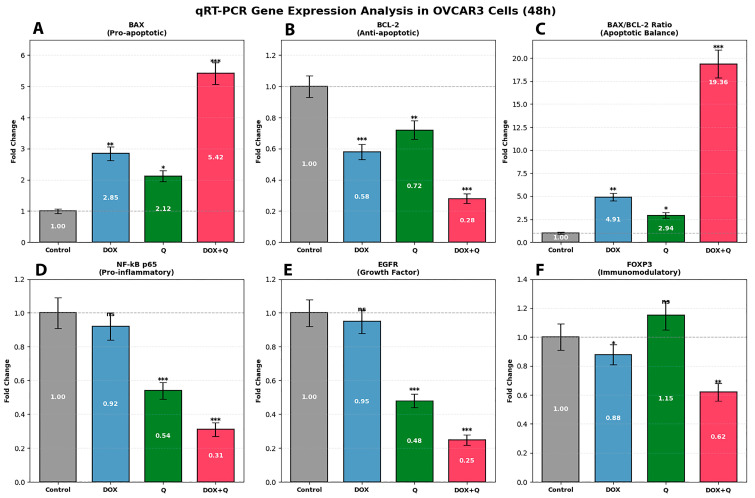
Effects of DOX, Q, and their combination on mRNA expression profiles in OVCAR3 cells. OVCAR3 cells were treated with DOX, Q, or their combination at concentrations corresponding to their 48 h IC_50_ values for 48 h. Relative mRNA expression levels were determined by RT-qPCR and normalized to GAPDH. Data are presented as fold change relative to control (mean ± SD, n = 3). (**A**) BAX, (**B**) BCL-2, (**C**) BAX/BCL-2 ratio, (**D**) NF-κB p65, (**E**) EGFR, and (**F**) FOXP3. Statistical significance is indicated as ns, non-significant; * *p* < 0.05, ** *p* < 0.01, and *** *p* < 0.001 versus control (one-way ANOVA followed by Tukey’s post hoc test).

RT-qPCR analysis was performed after 48 h treatment with DOX, Q, and the DOX+Q combination to determine relative mRNA expression levels of BAX, BCL-2, NF-κB p65, EGFR, and FOXP3 (Figure 10).

Compared with the control group, BAX expression increased to 2.85-fold following DOX treatment and to 2.12-fold following Q treatment. The highest increase was observed in the combination group, where BAX expression reached 5.42-fold.

In contrast, expression of the anti-apoptotic gene BCL-2 was reduced to 0.58-fold in DOX-treated cells and to 0.72-fold in Q-treated cells. A more substantial decrease was detected in the DOX+Q group, where expression declined to 0.28-fold. Consistent with these findings, the BAX/BCL-2 ratio increased markedly, reaching 4.91-fold in the DOX group, 2.94-fold in the Q group, and 19.36-fold following combination treatment.

NF-κB p65 expression remained largely unchanged after DOX exposure (0.92-fold), whereas Q treatment reduced expression to 0.54-fold. The lowest NF-κB p65 expression level was detected in the combination group (0.31-fold).

A similar pattern was observed for EGFR expression. While DOX treatment produced minimal alteration (0.95-fold), Q exposure reduced EGFR expression to 0.48-fold. Combined treatment with DOX and Q resulted in a further decrease to 0.25-fold.

FOXP3 expression demonstrated variable alterations among treatment groups, with expression levels measured as 0.88-fold in DOX-treated cells, 1.15-fold in Q-treated cells, and 0.62-fold in the DOX+Q group.

Taken together, combination treatment was associated with increased BAX expression together with reduced BCL-2, NF-κB p65, and EGFR expression levels relative to single-agent treatments.

### 3.10. Network Pharmacology and Bioinformatics Analysis Findings

#### 3.10.1. Target Gene Identification and Intersection Analysis

Potential target genes related to DOX and Q were collected from multiple databases and subsequently compared with ovarian cancer–associated genes after duplicate elimination and standardization of gene symbols.

Venn diagram analysis demonstrated overlapping gene clusters among DOX-associated targets, Q-associated targets, and ovarian cancer–related genes (Supplementary Figure S1). Eight genes were identified as common to all three datasets, representing the shared intersection between drug-related targets and disease-associated genes. These intersecting genes were regarded as potential targets associated with the DOX+Q combination in ovarian cancer.

#### 3.10.2. Protein–Protein Interaction Network Analysis

A PPI network was generated from the overlapping genes identified in the intersection analysis using the STRING database with a high-confidence interaction threshold (≥0.700) (Supplementary Figure S2).

Analysis of the generated network demonstrated multiple interactions among the identified genes, with several nodes exhibiting comparatively greater connectivity than others. Topological evaluation performed with the CytoHubba plugin identified AKT1, TP53, CASP3, CASP9, BCL2, and MAPK1 as highly connected hub nodes within the network.

Relative to the remaining nodes, these genes displayed higher degree and centrality scores, suggesting potential importance within the interaction network. Overall, the constructed PPI network offers a systems-level representation of the interactions among the identified targets.

#### 3.10.3. Gene Ontology Enrichment Analysis

GO enrichment analysis was conducted to define the functional characteristics of the overlapping target genes identified through network pharmacology analysis (Supplementary Figure S3).

Within the BP category, significantly enriched terms included regulation of apoptotic processes, cellular proliferation, oxidative stress response, DNA damage response, signal transduction, and immune-related responses.

For the MF category, enriched functional terms comprised protein kinase activity, ATP binding, enzyme binding, DNA binding, transcription factor activity, and ubiquitin ligase activity.

In the CC category, the majority of identified genes were associated with the cytoplasm, nucleus, mitochondrion, cytosol, plasma membrane, and endoplasmic reticulum. Overall, GO enrichment analysis provided a broad functional profile of the biological processes, molecular functions, and cellular localization patterns linked to the identified target genes.

#### 3.10.4. KEGG Pathway Enrichment Analysis

KEGG pathway enrichment analysis was conducted to determine signaling pathways associated with the overlapping target genes (Supplementary Figure S4).

Among the significantly enriched pathways were the PI3K–Akt signaling pathway, p53 signaling pathway, MAPK signaling pathway, apoptosis, pathways in cancer, cell cycle, FoxO signaling pathway, and NF-κB signaling pathway. All reported pathways satisfied the predefined significance criteria (*p* < 0.05 and FDR < 0.05). Overall, KEGG pathway analysis provided a general overview of signaling pathways linked to the identified target genes.

## 4. Discussion

The present study comprehensively investigated the combined effects of DOX and Q in ovarian cancer through integrated experimental and in silico analyses. Overall, the findings indicate that combined DOX+Q treatment enhances cytotoxic and pro-apoptotic responses in OVCAR3 cells while exerting a comparatively lower effect on non-tumoral HaCaT cells. Consistent findings obtained from viability assays, apoptosis analyses, oxidative stress measurements, gene expression profiling, and network-based bioinformatics analyses support the presence of a coordinated multi-level cellular response.

In OVCAR3 cells, DOX produced a clear dose- and time-dependent reduction in viability, consistent with its known mechanisms involving DNA intercalation and topoisomerase II inhibition [21,22]. By contrast, the higher IC_50_ values observed in HaCaT cells suggest relatively greater selectivity toward tumor cells. Q alone exerted limited cytotoxic activity, particularly during shorter exposure periods; however, prolonged treatment increased its efficacy, potentially due to intracellular accumulation and redox-associated effects [23]. Previous investigations examining combined DOX and Q treatment in different cancer models have reported variable outcomes depending on cellular context. In liver cancer models, Q enhanced DOX-mediated apoptosis through p53-dependent mechanisms together with modulation of Bcl-xl expression [24]. Similarly, studies in breast cancer cells demonstrated that Q may sensitize DOX-resistant cells to chemotherapy [25]. Conversely, some studies suggested that under certain conditions Q may attenuate ROS-mediated cytotoxicity induced by DOX, emphasizing the importance of concentration, cell type, and redox status [26]. In ovarian cancer models, Q-containing combinations have been associated with suppression of PI3K/Akt, STAT3, and NF-κB signaling pathways, thereby contributing to enhanced apoptosis and cytotoxicity [27].

The NAC rescue experiments provided additional insight into the involvement of oxidative stress in the observed cellular responses. NAC pretreatment partially restored cell viability while significantly decreasing ROS accumulation, suggesting that ROS contributes to, but does not completely explain, the cytotoxic effects induced by the combination treatment. The incomplete reversal indicates that additional mechanisms, including direct DNA damage and modulation of signaling pathways, may also participate in the observed response, consistent with the multifactorial nature of DOX-mediated cytotoxicity [24,26]. It should additionally be noted that the biological activity of NAC is not restricted solely to ROS scavenging. Previous reports demonstrated that NAC may influence multiple cellular processes through restoration of intracellular glutathione levels, modulation of thiol-disulfide balance, reduction in protein disulfide bonds, and regulation of redox-sensitive signaling pathways. Furthermore, NAC has been shown to affect protein function and cellular signaling independently of direct antioxidant activity, suggesting that the rescue effects observed in the present study may reflect broader redox-regulatory mechanisms rather than isolated ROS neutralization [28,29]. Therefore, although NAC pretreatment supports the involvement of oxidative stress in the observed cellular responses, these findings do not establish ROS as the sole mechanism responsible for DOX+Q-induced cytotoxicity.

CI analysis demonstrated a synergistic interaction between DOX and Q (CI < 1), particularly at lower DOX concentrations. This observation suggests that combination-based approaches may permit dose reduction in DOX while preserving therapeutic efficacy, an important consideration given the dose-dependent toxicity profile of DOX, especially cardiotoxicity [30]. Comparable findings have been described in previous studies showing that flavonoid-based combinations may improve the therapeutic index of conventional chemotherapeutic agents [31].

Apoptosis analyses revealed that combined treatment was associated with a pronounced increase in apoptotic cell populations together with elevated caspase-3 and caspase-9 activities. Immunocytochemical findings demonstrating increased caspase-9 expression further supported these observations. Concurrently, ROS analyses showed substantial intracellular oxidative stress elevation following combination treatment, which was markedly attenuated after NAC pretreatment. Collectively, these findings suggest that oxidative stress represents an important contributing factor in the apoptotic responses observed in the present study [32].

Gene expression profiling provided further molecular insight into the observed effects. Increased BAX expression together with suppression of BCL-2 and marked elevation of the BAX/BCL-2 ratio were consistent with activation of pro-apoptotic signaling mechanisms. Moreover, decreased NF-κB p65 and EGFR expression levels suggest modulation of inflammatory and proliferative signaling pathways. Reduced FOXP3 expression was also detected after DOX+Q treatment. Although the precise functional role of FOXP3 in ovarian cancer cells remains context-dependent and incompletely understood, previous studies have implicated FOXP3 in survival-associated and tumor-related immunomodulatory signaling processes. Accordingly, the observed reduction in FOXP3 expression may reflect alterations in regulatory pathways associated with tumor-related signaling and cellular adaptation responses. Nevertheless, these observations should be interpreted cautiously and require additional mechanistic validation. Furthermore, although the findings support involvement of multiple signaling pathways, mRNA-level alterations do not necessarily correspond directly to protein activity or functional pathway regulation [33].

Network pharmacology analyses further complemented the experimental data by identifying interconnected target genes and enriched signaling pathways. Detection of genes including TP53, AKT1, CASP3, CASP9, and BCL2 within the interaction network, together with enrichment of PI3K–Akt, MAPK, and p53 signaling pathways, suggests that the observed cellular responses may involve coordinated regulation across multiple signaling pathways. However, these observations should be regarded as hypothesis-generating and require additional experimental confirmation [34]. Although the number of overlapping targets identified through network pharmacology analysis was relatively limited, the identified genes included biologically important regulators associated with apoptosis, oxidative stress, and survival signaling pathways. Therefore, the bioinformatics findings should be interpreted as exploratory observations rather than definitive mechanistic evidence.

**Figure 11:**
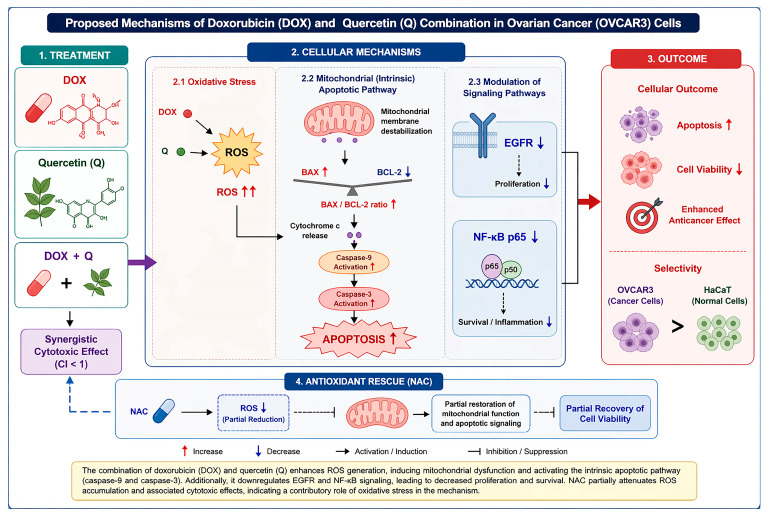
Schematic representation of cellular responses associated with combined DOX and Q treatment in OVCAR3 cells. The diagram illustrates the proposed interactions between oxidative stress, mitochondrial signaling, and regulatory pathways following combination treatment. Increased intracellular ROS levels are linked to alterations in mitochondrial apoptotic balance, including modulation of BAX and BCL-2 and signaling events associated with caspase-9 and caspase-3 activation. In addition, changes in EGFR- and NF-κB-related signaling are depicted, reflecting potential effects on proliferation and survival pathways. The modulatory effect of NAC on ROS levels is also indicated, consistent with a modulatory role in treatment-associated cellular responses. Red double upward arrows (↑↑) indicate a marked increase or enhancement. Red downward arrows (↓) indicate a decrease or reduction. Black solid arrows indicate activation, induction, or progression of a biological process, whereas black dashed arrows indicate an indirect or putative association.

Integration of the experimental findings with network-based analyses suggests that combined DOX and Q treatment induces a coordinated multi-level cellular response in OVCAR3 cells. One prominent feature of this response appears to be substantial intracellular ROS accumulation, which may function as an upstream regulator of downstream apoptotic signaling. Elevated ROS levels were associated with disruption of mitochondrial homeostasis, reflected by increased BAX/BCL-2 ratios together with subsequent activation of caspase-9 and caspase-3, indicating engagement of mitochondria-associated apoptotic pathways. Simultaneously, reduced EGFR and NF-κB p65 expression suggests attenuation of proliferative and survival-associated signaling networks. Taken together, these findings indicate that the cytotoxic activity of the DOX+Q combination is unlikely to depend on a single dominant mechanism, but rather on the convergence of oxidative stress, apoptotic signaling, and modulation of key regulatory pathways. The NAC rescue experiments further support this interpretation. Partial reduction in ROS levels and associated cellular responses after NAC pretreatment suggests that oxidative stress contributes substantially to the observed cytotoxicity but does not fully account for it. This incomplete reversibility supports a model in which ROS functions as an important, though not exclusive, mediator of combination-induced cell death, while additional ROS-independent mechanisms may also participate in the integrated response. Overall, the present findings support a mechanistic framework in which co-treatment with DOX and Q disrupts redox balance, mitochondrial integrity, and signaling homeostasis in a coordinated manner, ultimately promoting enhanced apoptotic cell death in ovarian cancer cells (Figure 11).

Several limitations of the present study should be taken into consideration. First, experiments were performed using only a single ovarian cancer cell line (OVCAR3), which may restrict the broader applicability of the findings across different molecular subtypes of ovarian cancer. Therefore, further validation using additional cell lines, including drug-resistant and genetically heterogeneous models, is warranted. Second, the analyses were predominantly conducted at the mRNA level, whereas protein-level confirmation using methods such as Western blotting was not performed and should be considered an important limitation. Specifically, proteins associated with mitochondrial apoptosis, including cleaved caspase-3, cleaved caspase-9, PARP, and cytochrome c, were not evaluated. Third, mechanistic interpretation remains limited because functional validation studies targeting specific pathways, such as pathway inhibition or gene silencing experiments, were not included. Furthermore, although MTT-based viability assays are commonly applied in anticancer studies, such assays may be affected by alterations in mitochondrial function and cellular redox metabolism, particularly when redox-active compounds such as DOX and Q are investigated. For this reason, synergistic effects observed in the present study were interpreted together with complementary analyses including apoptosis assessment, caspase activity measurements, ROS evaluation, immunocytochemical staining, and gene expression profiling. Nevertheless, confirmation of the reported synergistic effects would be strengthened by additional orthogonal viability and cytotoxicity assays. Another important limitation is the lack of in vivo experiments, which prevents evaluation of pharmacokinetic properties, interactions within the tumor microenvironment, and systemic toxicities such as DOX-associated cardiotoxicity. In addition, although HaCaT cells were utilized as a non-tumoral model, they do not fully represent normal ovarian tissue biology. Inclusion of additional normal cell models would therefore improve translational relevance. Moreover, ROS and apoptosis analyses following combination treatment were not conducted in HaCaT cells. Consequently, conclusions regarding selective toxicity should be regarded as preliminary and require confirmation using additional non-tumoral cell models together with complementary functional analyses. Finally, because several flow cytometry–based assays relied on FITC-associated fluorescence detection, possible interference arising from the intrinsic fluorescence characteristics of DOX cannot be entirely excluded despite the use of compensation procedures and appropriate control settings. Accordingly, ROS- and apoptosis-related findings should be interpreted with methodological caution.

Future investigations should address these limitations through incorporation of multiple ovarian cancer cell models, protein-level validation studies, functional mechanistic assays, and in vivo experimental systems to more clearly define the therapeutic potential and safety profile of the DOX+Q combination.

## 5. Conclusions

In conclusion, the findings of the present study suggest that combined treatment with DOX and Q enhances cytotoxic and apoptotic responses in OVCAR3 ovarian cancer cells, while comparatively lower sensitivity was observed in non-tumoral HaCaT cells under the current experimental conditions. These effects were associated with elevated intracellular ROS accumulation, activation of mitochondria-related apoptotic signaling pathways, and modulation of regulatory pathways including EGFR and NF-κB.

The partial attenuation of these effects following NAC pretreatment indicates that oxidative stress contributes to the coordinated cellular response, although it does not appear to represent the sole underlying mechanism, supporting a multifactorial mode of action.

Although the results indicate that the DOX+Q combination is associated with coordinated regulation of redox homeostasis, apoptotic signaling, and proliferative pathways, interpretation of these findings should remain cautious because the study was limited to in vitro experiments and lacked protein-level as well as in vivo validation.

Overall, the present study provides a mechanistic framework that may guide future investigations evaluating combination-based therapeutic strategies in ovarian cancer, particularly in relation to improving therapeutic efficacy while investigating treatment-associated selectivity tendencies.

## Figures and Tables

**Table 1:** Primer sequences used in qRT-PCR analysis.

Genes	Primer Sequence (5′→3′)
*BAX*	GGGGACGAACTGGACAGTAA, CAGTTGAAGTTGCCGTCAGA
*BCL-2*	ATGTGTGTGGAGAGCGTCAA, ACAGTTCCACAAAGGCATCC
*NF-κB p65 (RELA)*	GCGATGGCTTCTATGAGGCT, TGCTTCTGTCCCCTTCTTCC
*EGFR*	AGGCACGAGTAACAAGCTCAC, ATGAGGACATAACCAGCCACC
*FOXP3*	CAGCACATTCCCAGAGTTCATC, AGTGAGGTCGGTAAGAAGCAG
*GAPDH*	ACCCACTCCTCCACCTTTGA, CTGTTGCTGTAGCCAAATTCGT

## Data Availability

The original contributions presented in this study are included in the article. Further inquiries can be directed at the corresponding author.

## Supplementary Materials

The following supporting information can be downloaded at: https://www.mdpi.com/article/10.3390/biomedicines14061248/s1, Figure S1: Identification of Common Targets of DOX, Q, and Ovarian Cancer; Figure S2: Protein–Protein Interaction Network of Hub Genes; Figure S3: GO Enrichment Analysis; Figure S4: KEGG Pathway Enrichment Analysis; Table S1: Summary of Flow Cytometry ROS Analysis Parameters in OVCAR3 Cells.

## Abbreviations

BAX	Bcl-2-associated X protein
BCL-2	B-cell lymphoma 2
BP	Biological Process
CC	Cellular Component
CI	Combination Index
DAB	3,3′-Diaminobenzidine
DCFH-DA	2′,7′-Dichlorodihydrofluorescein diacetate
DMEM	Dulbecco’s Modified Eagle Medium
DOX	Doxorubicin
EGFR	Epidermal Growth Factor Receptor
ELISA	Enzyme-Linked Immunosorbent Assay
FBS	Fetal Bovine Serum
FDR	False Discovery Rate
FITC	Fluorescein Isothiocyanate
FOXP3	Forkhead Box P3
GO	Gene Ontology
IC_50_	Half maximal inhibitory concentration
KEGG	Kyoto Encyclopedia of Genes and Genomes
MAPK	Mitogen-Activated Protein Kinase
MF	Molecular Function
MTT	3-(4,5-dimethylthiazol-2-yl)-2,5-diphenyltetrazolium bromide
NAC	N-acetyl-L-cysteine
NF-κB	Nuclear Factor kappa B
PBS	Phosphate-Buffered Saline
PI	Propidium Iodide
PPI	Protein–Protein Interaction
Q	Quercetin
qRT-PCR	Quantitative Real-Time Polymerase Chain Reaction
ROS	Reactive Oxygen Species
RPMI	Roswell Park Memorial Institute medium
SD	Standard Deviation
STRING	Search Tool for the Retrieval of Interacting Genes/Proteins
