# Supplementary material for: Synergistic Effects of Doxorubicin and Quercetin on ROS-Associated Apoptosis and EGFR/FOXP3 Modulation in OVCAR3 Cells
Source: Biomedicines. 2026 May 30;14(6):1248. doi: 10.3390/biomedicines14061248 (PMC13297588; doi:10.3390/biomedicines14061248)
Supplement: Supplementary file 1 [file biomedicines-14-01248-s001.zip › Supplementary Table S1.pdf]

**Table S1.** Summary of Flow Cytometry ROS Analysis Parameters in OVCAR3 Cells

| Category | Parameter / Comparison | Value / Observation | Interpretation / Purpose |
|---|---|---|---|
| ***Raw Fluorescence Intensity (MFI)*** | Unstained Control | 120 | Autofluorescence baseline |
| | Control | 250 (1.00-fold) | Basal ROS level |
| | Q | 545 (2.18-fold) | Moderate ROS increase |
| | DOX | 860 (3.45-fold) | Significant ROS induction |
| | DOX+Q | 1705 (6.82-fold) | Strong synergistic ROS increase |
| | NAC + DOX+Q | 380 (1.52-fold) | ROS suppression/rescue effect |
| ***ROS-Positive Cell Distribution*** | Unstained Control | 0.5% ROS-positive / 99.5% ROS-negative | Background fluorescence |
| | Control | 3% ROS-positive / 97% ROS-negative | Basal ROS distribution |
| | Q | 18% ROS-positive / 82% ROS-negative | Moderate oxidative response |
| | DOX | 35% ROS-positive / 65% ROS-negative | Increased oxidative stress |
| | DOX+Q | 72% ROS-positive / 28% ROS-negative | Markedly elevated ROS accumulation |
| | NAC + DOX+Q | 10% ROS-positive / 90% ROS-negative | NAC-mediated ROS attenuation |
| ***Flow Cytometry Controls*** | FMO-FITC | MFI ≈ 140; 1–2% positive | Negative gate determination |
| | FITC Single-Stained | MFI ≈ 900–1500; 95–99% positive | Compensation setup |
| | Unstained Cells | MFI ≈ 120; <1% positive | Autofluorescence reference |
| ***Compensation Matrix*** | FITC Spillover | PE: 4%; PerCP: 0.5%; APC: 0% | Spectral compensation |
| | PE Spillover | PerCP: 1% | Spectral compensation |
| | PerCP Spillover | APC: 0.5% | Spectral compensation |
| ***Cytometer Acquisition Parameters*** | ROS Dye | DCFH-DA | ROS detection |
| | Excitation Laser | 488 nm | FITC excitation |
| | Detection Channel | FITC (530/30 nm) | Fluorescence acquisition |
| | Events Acquired | 10,000–20,000 cells | Statistical reliability |
| | FSC Threshold | 50,000–70,000 | Debris exclusion |
| | FITC PMT Voltage | 420–500 V | Signal optimization |
| | Compensation Requirement | Minimal | Low spectral overlap |
| | Gating Strategy | FSC/SSC → Singlets → FITC-positive | Population selection |
| | ROS Positive Threshold | ~$10^3$ fluorescence units | Positive event definition |
| ***Statistical Annotation Mapping*** | ns | $p > 0.05$ | Non-significant |
| | * | $p < 0.05$ | Significant |
| | ** | $p < 0.01$ | Highly significant |
| | *** | $p < 0.001$ | Very highly significant |

| Category | Parameter / Comparison | Value / Observation | Interpretation / Purpose |
|---|---|---|---|
| ***Biological Interpretation Summary*** | Q vs Control | — | Quercetin moderately increased intracellular ROS |
| | DOX vs Control | — | Doxorubicin significantly induced oxidative stress |
| | DOX+Q vs DOX | — | Combination treatment markedly enhanced ROS production |
| | NAC + DOX+Q vs DOX+Q | — | NAC substantially attenuated ROS accumulation |
| | NAC + DOX+Q vs Control | — | ROS levels partially returned toward baseline |
