# Supplementary figures and images for: Synergistic Effects of Doxorubicin and Quercetin on ROS-Associated Apoptosis and EGFR/FOXP3 Modulation in OVCAR3 Cells
Source: Biomedicines. 2026 May 30;14(6):1248. doi: 10.3390/biomedicines14061248 (PMC13297588; doi:10.3390/biomedicines14061248)

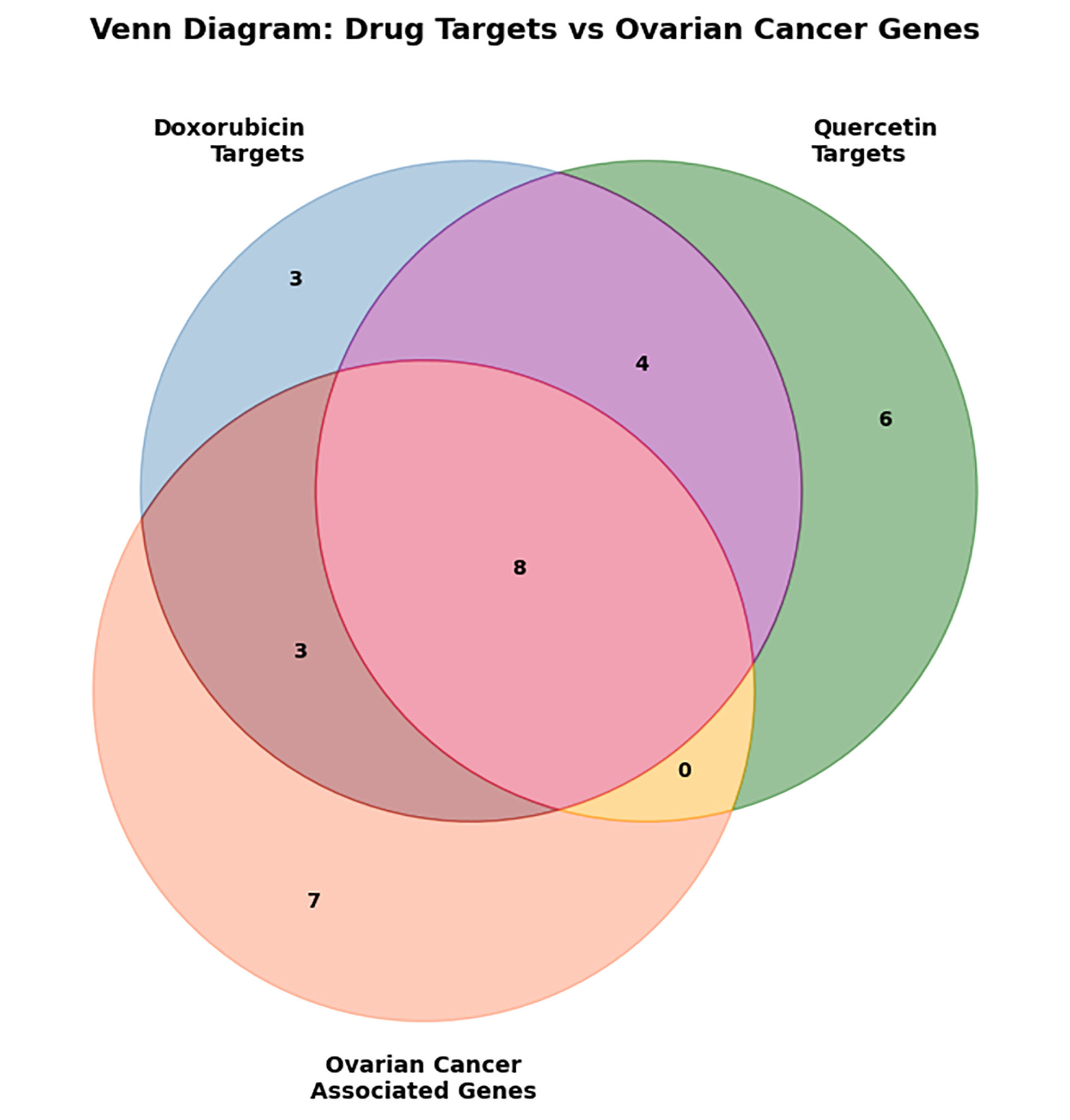

Supplement: Supplementary file 1 [file biomedicines-14-01248-s001.zip › Supplementary Figure S1.jpg]

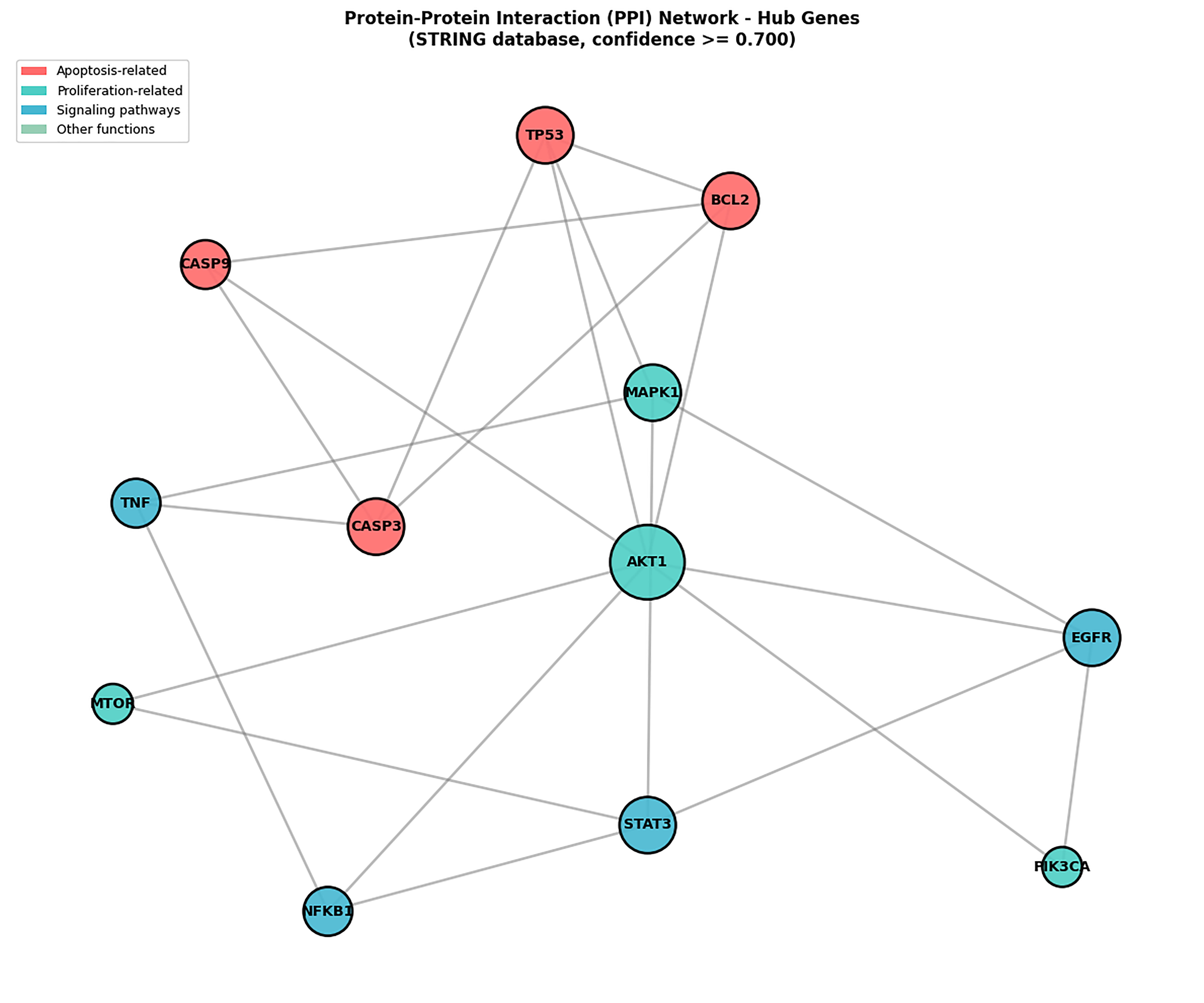

Supplement: Supplementary file 1 [file biomedicines-14-01248-s001.zip › Supplementary Figure S2.jpg]

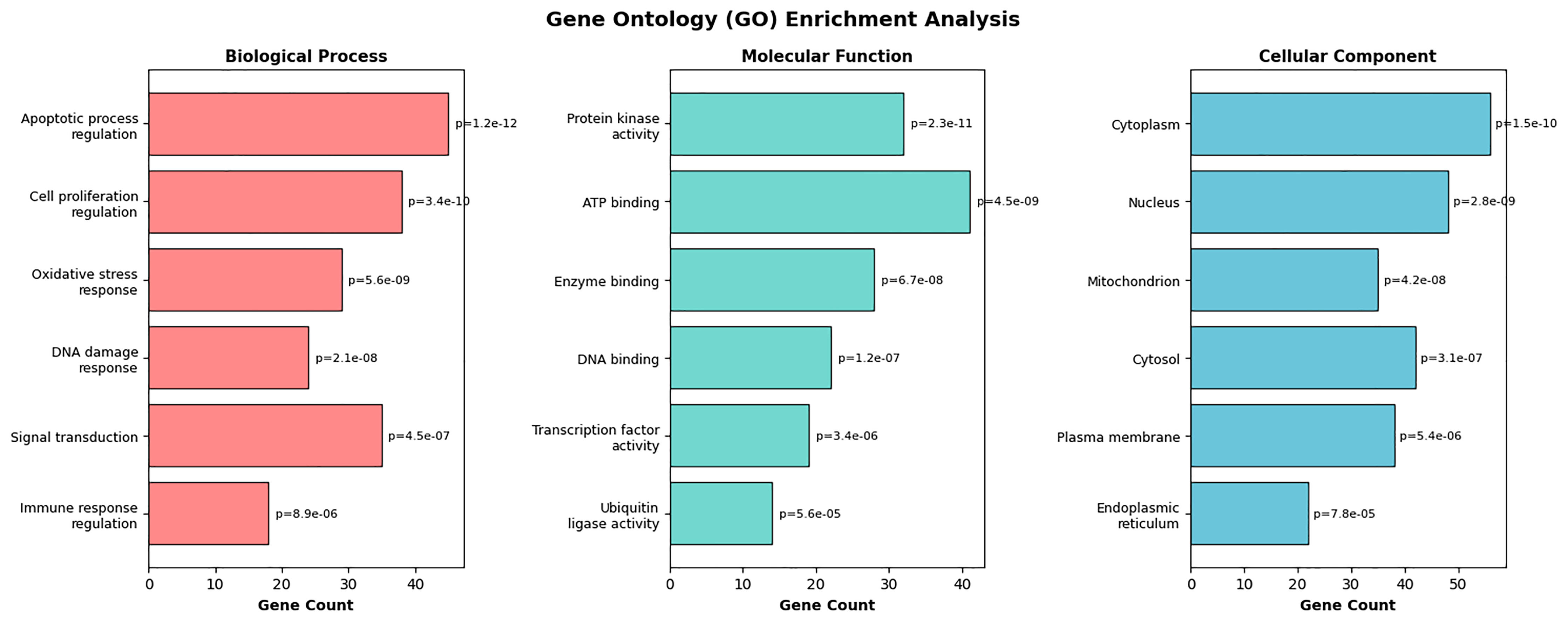

Supplement: Supplementary file 1 [file biomedicines-14-01248-s001.zip › Supplementary Figure S3.jpg]

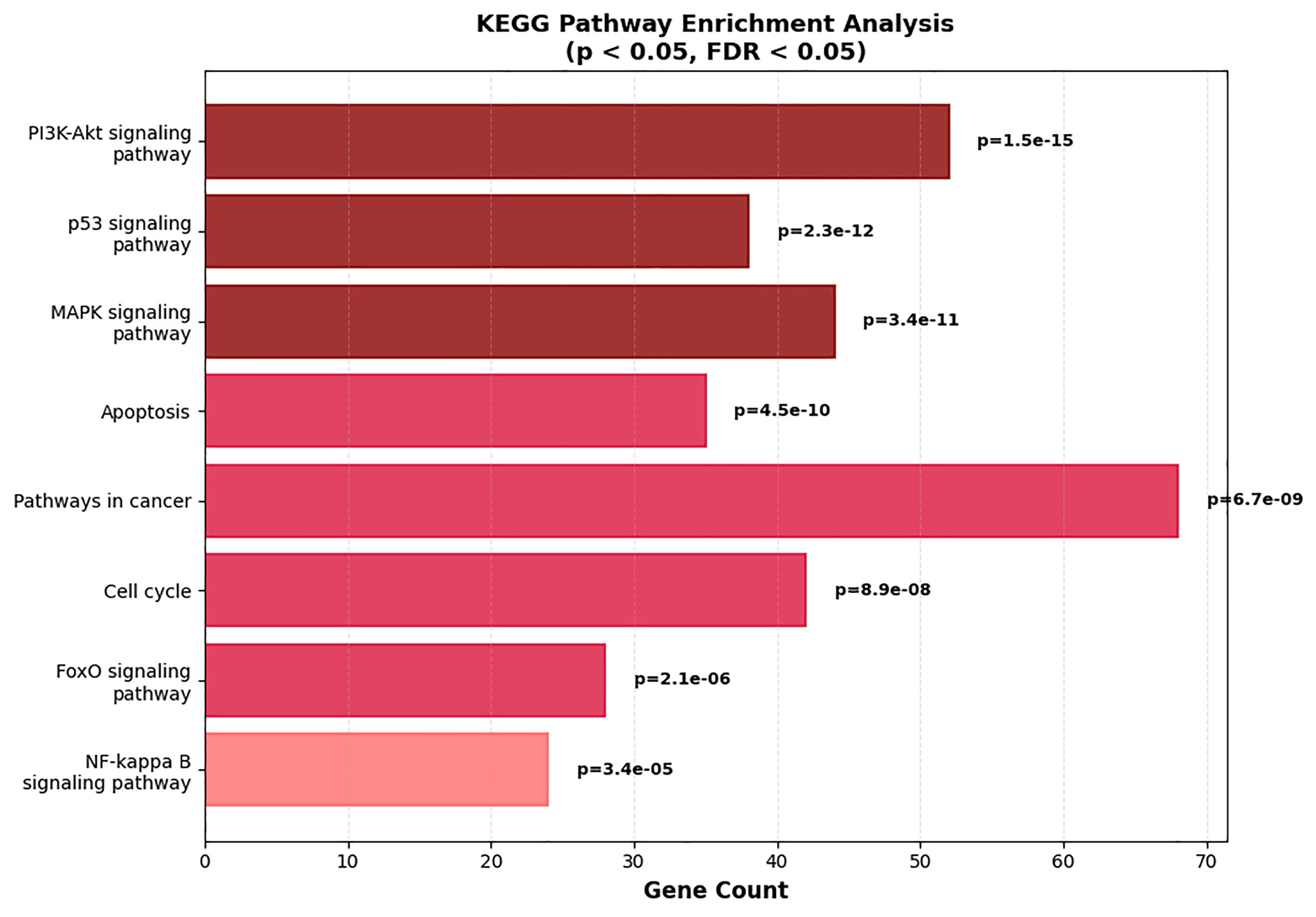

Supplement: Supplementary file 1 [file biomedicines-14-01248-s001.zip › Supplementary Figure S4.jpg]
